# Type I interferon biomarker in idiopathic inflammatory myopathies: associations of Siglec-1 with disease activity and treatment response

**DOI:** 10.1093/rheumatology/keae630

**Published:** 2024-11-19

**Authors:** Renske G Kamperman, Saskia R Veldkamp, Sanne W Evers, Johan Lim, Ivo van Schaik, Annet van Royen-Kerkhof, Femke van Wijk, Anneke J van der Kooi, Marc Jansen, Joost Raaphorst

**Affiliations:** Department of Neurology, Amsterdam University Medical Centre, Location AMC, Amsterdam, The Netherlands; Center for Translational Immunology, University Medical Center Utrecht, Utrecht, The Netherlands; Department of Neurology, Amsterdam University Medical Centre, Location AMC, Amsterdam, The Netherlands; Department of Rehabilitation Medicine, Amsterdam University Medical Centre, Location AMC, Amsterdam, The Netherlands; Department of Neurology, Amsterdam University Medical Centre, Location AMC, Amsterdam, The Netherlands; Sanquin, Amsterdam, The Netherlands; Department of Pediatric Rheumatology and Immunology, Wilhelmina Children’s Hospital, University Medical Center Utrecht, Utrecht, The Netherlands; Center for Translational Immunology, University Medical Center Utrecht, Utrecht, The Netherlands; Department of Neurology, Amsterdam University Medical Centre, Location AMC, Amsterdam, The Netherlands; Department of Pediatric Rheumatology and Immunology, Wilhelmina Children’s Hospital, University Medical Center Utrecht, Utrecht, The Netherlands; Department of Neurology, Amsterdam University Medical Centre, Location AMC, Amsterdam, The Netherlands

**Keywords:** Siglec-1, blood-based biomarker, idiopathic inflammatory myopathy, dermatomyositis, immune-mediated necrotizing myopathy, myositis, type I interferon, disease activity, treatment response

## Abstract

**Objectives:**

Novel biomarkers are needed to guide therapy in idiopathic inflammatory myopathies (IIM). Expression of Siglec-1, a type I interferon biomarker, was examined in adult patients with IIM in relation to disease activity and treatment response.

**Methods:**

We analyzed PBMC samples from 19 newly diagnosed adult IIM patients who participated in a phase-2 pilot study on efficacy of intravenous immunoglobulin (IVIG) monotherapy, and from 9 healthy controls. Siglec-1 expression on monocytes was measured by flow cytometry before and after treatment, and was evaluated in relation to IIM subtype, physician global activity (PhGA) scores, manual muscle strength (MMT) and the total improvement score (TIS).

**Results:**

Diagnoses included dermatomyositis (DM; *n* = 9), immune-mediated necrotizing myopathy (IMNM; *n* = 5), non-specific/overlap myositis (NSM/OM; *n* = 4) and antisynthetase syndrome (ASyS; *n* = 1). All patients showed increased Siglec-1 expression at baseline. Relative median fluorescence intensity of Siglec-1 was highest in patients with DM. After 9 weeks, follow-up samples were available for 15 patients of whom 10 patients showed a decline in Siglec-1 expression. In DM, Siglec-1 correlated with disease activity (MMT; rs = −0.603, *P* = 0.013 and PhGA; rs = 0.783, *P* < 0.001) and with the TIS (rs = −0.786, *P* = 0.036).

**Conclusion:**

Siglec-1 was increased in treatment-naive IIM patients and showed a decline after IVIG monotherapy. In DM, Siglec-1 expression correlated with relevant clinical measures. This underlines the dynamic role of type I IFN in IIM and the biomarker potential of Siglec-1, in particular in DM. These findings should be further validated in larger cohorts with longer follow-up.

Rheumatology key messages

- Elevated Siglec-1 in treatment-naive patients with idiopathic inflammatory myopathies (IIM) declined after IVIG monotherapy.
- Decline of Siglec-1 correlated with clinical improvement in patients with dermatomyositis.
- Siglec-1 emerges as a reliable biomarker in IIM, in particular dermatomyositis.

## Introduction

Idiopathic inflammatory myopathies (IIM), commonly referred to as ‘myositis’, are a group of heterogeneous autoimmune disorders characterized by subacute-onset and often severe, progressive proximal muscle weakness [1]. The most prevalent subtypes are dermatomyositis (DM), antisynthetase syndrome (ASyS), immune-mediated necrotizing myopathy (IMNM) and non-specific/overlap myositis (NSM/OM).

Accurate assessment of disease activity in IIM is crucial for initiating and maintaining the most effective immunosuppressive treatment. Clinical signs (e.g. muscle weakness) and serum creatine kinase (sCK) are useful markers of disease activity, but interpretation can be difficult (due to pain, fatigue, disease damage) and sCK can be normal in patients with IIM, mostly in patients with DM [2, 3]. Therefore, there is a need for a more sensitive and responsive marker of disease activity in IIM.

Activation of the type I interferon (IFN-I) pathway is thought to be an important feature of the pathogenesis of DM, since increased IFN-stimulated gene (ISG) expression has been demonstrated in muscle, blood and skin tissue and the presence of IFN-I-inducible myxovirus resistance protein A (MxA) was detected in perifascicular myofibers in muscle biopsies [4–7]. Among IIM subtypes, the IFN-I pathway was shown to be predominantly activated in (juvenile) DM and to a lesser extent in ASyS and NSM/OM, but not in IMNM, although data of the latter subgroup is scarce [6, 7]. A reliable marker reflecting IFN-I pathway activation would allow for patient stratification in terms of targeted treatment and monitoring of treatment response, as well as disease activity [8–11].

Direct quantification of IFN-I, which includes IFN-α and -β, has proven difficult due to low circulating levels. Indirect assessment of IFN-I activity by measuring ISG expression levels [12, 13] has significantly contributed to our understanding of IFN-I pathway involvement in DM, but is relatively time-consuming, limiting its clinical applicability. Among blood-based biomarkers induced by IFN-I, Sialic acid-binding Ig-like lectin (Siglec-1) is one of the most responsive ones. Increased Siglec-1 expression has been observed in SLE [12, 14] and rheumatoid arthritis (RA) [15]. In patients with juvenile DM (JDM), increased levels of Siglec-1 expression on circulating monocytes reflected the type I IFN gene signature, correlated with clinical disease activity and identified a subgroup at risk for treatment intensification [16]. Current evidence on the role of Siglec-1 in *adult* IIM is promising but limited [17]. Here, we measured Siglec-1 on circulating monocytes in treatment-naive adult IIM patients before and shortly after immunomodulatory treatment (intravenous immunoglobulin (IVIG) monotherapy). Expression levels were correlated with clinical improvement and disease activity measures.

## Methods

### Participants

We used data from the IMMEDIATE study [18]. This prospective phase-2 open-label pilot study investigated the efficacy and safety of first-line IVIG monotherapy in IIM patients within a period of 9 weeks. In the IMMEDIATE study, 20 adult IIM patients were included between March 2017 and January 2019. All patients had biopsy-proven IIM based on the 2004 European Neuromuscular Centre (ENMC) criteria, with a disease duration of <9 months [19] and at least a moderate degree of muscle weakness (10% loss of power on manual muscle testing of 13 muscle groups). Immunohistochemical analysis of muscle biopsies included expression of MxA, an IFN-I-inducible protein, in perifascicular myofibers.

Additional inclusion criteria are described elsewhere [18]. Prior use of low-dose prednisone (<20 mg) and other immunosuppressive medication (e.g. methotrexate and azathioprine), was allowed if it was (i) stopped four weeks prior to inclusion, and (ii) duration was limited to 2 weeks (prednisone) or 4 weeks (other medication) and (iii) there was no improvement upon treatment.

Patients were treated with monotherapy IVIG with 3-week intervals; first course 2 g/kg; second and third course 1 g/kg. Patients with initial improvement after the loading dose but insufficient response as judged by an experienced clinician, received an additional dose 1 week later, as described previously [18]. Despite this, seven patients prematurely ended the study because of an insufficient response (none of the patients deteriorated) and switched to rescue medication.

Following the end-of-study visit, patients received standard treatment consisting of (combinations of) corticosteroids, steroid-sparing agents, rituximab, or IVIG, depending on the level of disease activity.

A control group consisted of nine healthy adults. The study was conducted with approval of the research protocol by the Medical Ethics Committee (METC) of the Academic Medical Centre, University of Amsterdam, the Netherlands and in accordance with the Declaration of Helsinki. The IMMEDIATE study was registered in the Netherlands Trial Register (Netherlands Trial Register identifier NTR6160). All patients gave written informed consent.

### Clinical parameters

Clinical data and peripheral blood mononuclear cells (PBMCs) were obtained at baseline (treatment-naïve) and at the end-of-study visit after treatment with IVIG monotherapy for 9 weeks or before the switch to rescue therapy. We assessed six measures of disease activity, so-called Core Set Measures (CSMs), which allow for calculation of the Total Improvement Score (TIS), which is a validated composite outcome measure of clinical improvement in IIM [20–22]. For correlation analyses with Siglec-1, we used three individual CSMs, based on previous studies [16, 17]:

1. Manual muscle testing (MMT-26) of 12 muscle groups bilaterally along with two axial muscle groups (total score ranges from 0 to 260 with higher scores indicating more strength) [22].
2. sCK, the most used conventional blood-based marker of disease activity in IIM, with an upper limit of normal of 171 U/l in our centre [23].
3. Physician’s global assessment (PhGA), a validated CSM assessed on a visual analogue scale (0–10, with higher scores indicating more overall disease activity). The assessment is done by a trained physician and is based on patient-reported disease activity, clinical manifestations and ancillary investigations [24].

At baseline and the end-of-study visit, three additional CSMs, i.e. Patient’s global assessment (PGA), extramuscular disease activity and the Health Assessment Questionnaire (HAQ), were assessed to calculate the Total Improvement Score (TIS) [20]. For the current study, we used the cut-off for moderate improvement (TIS ≥ 40 points) to define responders and non-responders, similar to our previous publication [18].

### Siglec-1 expression on monocytes

Cryopreserved PBMCs were thawed in a 37°C water bath. One million cells were stained with fixable viability dye eFluor 780 (Thermo Fisher Scientific) for 30 min at 4°C to determine cell viability by flow cytometry. Surface staining was subsequently performed with the antibodies V500-conjugated anti-human CD14 (clone M5E2, BD) and PE-conjugated anti-human Siglec-1 (CD169) (clone 7–239, Thermo Fisher Scientific) for 20 min at 4°C in PBS containing 2% fetal bovine serum (FBS), 0.1% sodium azide and 2% mouse serum. Measurement of Siglec-1 levels was performed using the BD LSRFortessa™ flow cytometer and further analyzed with FlowjoV10 software.

Expression of Siglec-1 on monocytes was expressed in (i) percentage of Siglec-1-positive cells within the live CD14+ monocyte population and (ii) relative median fluorescence intensity (rMFI), indicating the level of fluorescence signal within the live CD14+ monocyte population, relative to the median MFI in healthy controls, which was set at 100.

### Statistical analysis

Continuous data are presented as median and interquartile range (IQR: 25th–75th percentiles). Categorical data are presented as absolute numbers (percentage). Between-group differences of Siglec-1 expression were examined with the Kruskal–Wallis test with post-hoc Dunn’s test. Longitudinal changes of Siglec-1 expression were examined with the Wilcoxon signed rank test. Associations between Siglec-1 expression and clinical and histological parameters were examined with Spearman’s rank test (using change scores where appropriate) and by comparing groups with high and low Siglec-1 expression at baseline (based on median split). For correlation analyses, where appropriate, data from baseline and follow-up were combined, in order to optimize the power. A two-sided alpha level of 0.05 was considered statistically significant. Statistical analysis was performed using SPSS (IBM SPSS statistics version 28).

## Results

### Study population

**Figure 1.**
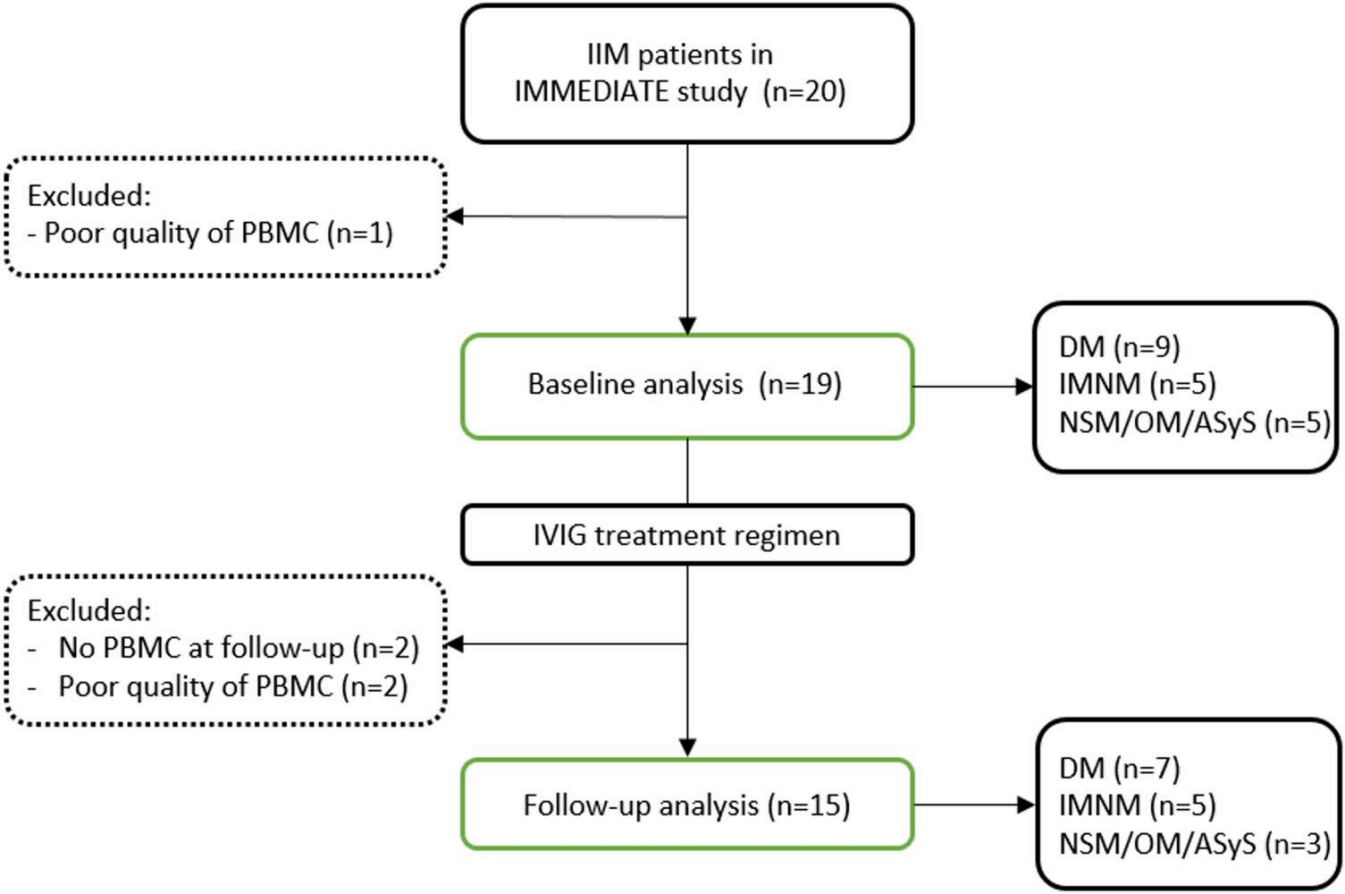
Flowchart. IIM, idiopathic inflammatory myopathy; PBMC, peripheral blood mononuclear cells; DM, dermatomyositis; IMNM, immune-mediated necrotizing myopathy; NSM/OM, non-specific/overlap myositis; ASyS, antisynthetase syndrome

We included 19 out of 20 patients for baseline analysis and 15 patients for longitudinal analysis (Fig. 1). One patient was excluded at baseline due to poor quality of PBMCs and four patients were excluded from follow-up analyses due to lack or poor quality of PBMCs. The baseline cohort consisted of patients (63% female) with DM (*n* = 9), IMNM (*n* = 5), NSM/OM (*n* = 4) and ASyS (*n* = 1) (Fig. 1, Table 1). Due to the small sample size, we analyzed NSM/OM/ASyS as one IIM subgroup. The follow-up cohort consisted of seven DM patients, five IMNM patients and three NSM/OM/ASyS patients. In four patients, follow-up visits including sampling were at 3 or 6 (instead of 9) weeks, due to insufficient response and a premature end-of-study visit [18].

**Table 1.** Baseline characteristics

	**Total IIM** ***n* = 19**	**DM** ***n* = 9**	**IMNM** ***n* = 5**	**NSM/OM/ASyS** ***n* = 5**	**HC** ***n* = 9**
Female, *n* (%)	12 (63)	5 (56)	3 (60)	4 (80)	5 (56)
Age in years, median (IQR)	56 (37–67)	47 (32–61)	65 (44–70)	64 (50–75)	39 (32–47)
Disease duration in months, median (IQR)	5 (4.0–6.0)	4 (3.5–5.5)	6 (2.0–6.5)	6.0 (4.5–9.0)	N/A
MMT-26, median (IQR)	212 (203–226)	222 (185–238)	210 (195–213)	220 (208–227)	N/A
PhGA, median (IQR)	3.7 (3.0–4.0)	3.4 (2.7–4.0)	3.8 (3.0–4.0)	3.7 (3.1–5.9)	N/A
Serum CK (U/l), median (IQR)	1080 (179–3177)	190 (103–2046)	10 353 (4727–20 273)	840 (373–2075)	N/A
Myositis-specific autoantibodies^a^, *n* (%)	12 (63.2)	7 (77.8)	4 (80.0)	1 (20.0)	N/A
Myositis-associated autoantibodies^a^, *n* (%)	4 (21.1)	1 (11.1)	1 (20.0)	2 (40.0)	N/A
Seronegative, *n* (%)	3 (15.8)	1 (11.1)	1 (20.0)	1 (20.0)	N/A
MxA positive in muscle biopsy, *n* (%)^b^	8 (50.0)	8 (88.9)	0 (0)	0 (0)	N/A

a Autoantibodies were considered absent when semiquantitative score was + (weakly positive) and present when scores were ++ (positive) and +++ (strongly positive). Myositis-specific autoantibodies present included anti-NXP2 (*n* = 3), anti-TIF1ƴ (*n* = 2), anti-MDA5 (*n* = 1), anti-SRP (*n* = 1), anti-HMGCR (*n* = 3), anti-Mi2 (*n* = 1), anti-Jo1 (*n* = 1).

b Muscle biopsies of 16 patients (84%) were eligible for staining with myxovirus resistance protein A (MxA). IIM, idiopathic inflammatory myopathies; DM, dermatomyositis; IMNM, immune-mediated necrotizing myopathies; NSM/OM, non-specific myositis/overlap myositis; ASyS, antisynthetase syndrome; MMT, manual muscle testing; PhGA, physician global activity; CK, creatine kinase; HC, healthy controls; IQR, interquartile range; N/A, not applicable.

Median time from disease onset to diagnosis was 5.0 months (IQR 4.0–6.0) and median age was 56 years (IQR 37–67). Two patients were treated with immunosuppressants prior to study enrolment; both stopped 1 month before inclusion; one patient was treated for 5 days with low dose prednisone and one patient was treated for 1 month with methotrexate. Median PhGA at baseline was 3.7 (IQR 3.0–4.0, Table 1). In five patients, extramuscular involvement (e.g. myocarditis or ILD) was present. Three patients with DM had normal CK values (below 170 U/l); three other patients with DM had values between 170 and 190 U/l. Two OM patients had mixed connective tissue disease (MCTD) and Sjögren’s syndrome, respectively. One patient in the IMNM group also had systemic sclerosis. Two patients were diagnosed with cancer of whom one patient was diagnosed with cancer between baseline and follow-up sampling.

### Siglec-1 expression in IIM and healthy controls at baseline and follow-up

**Figure 2.**
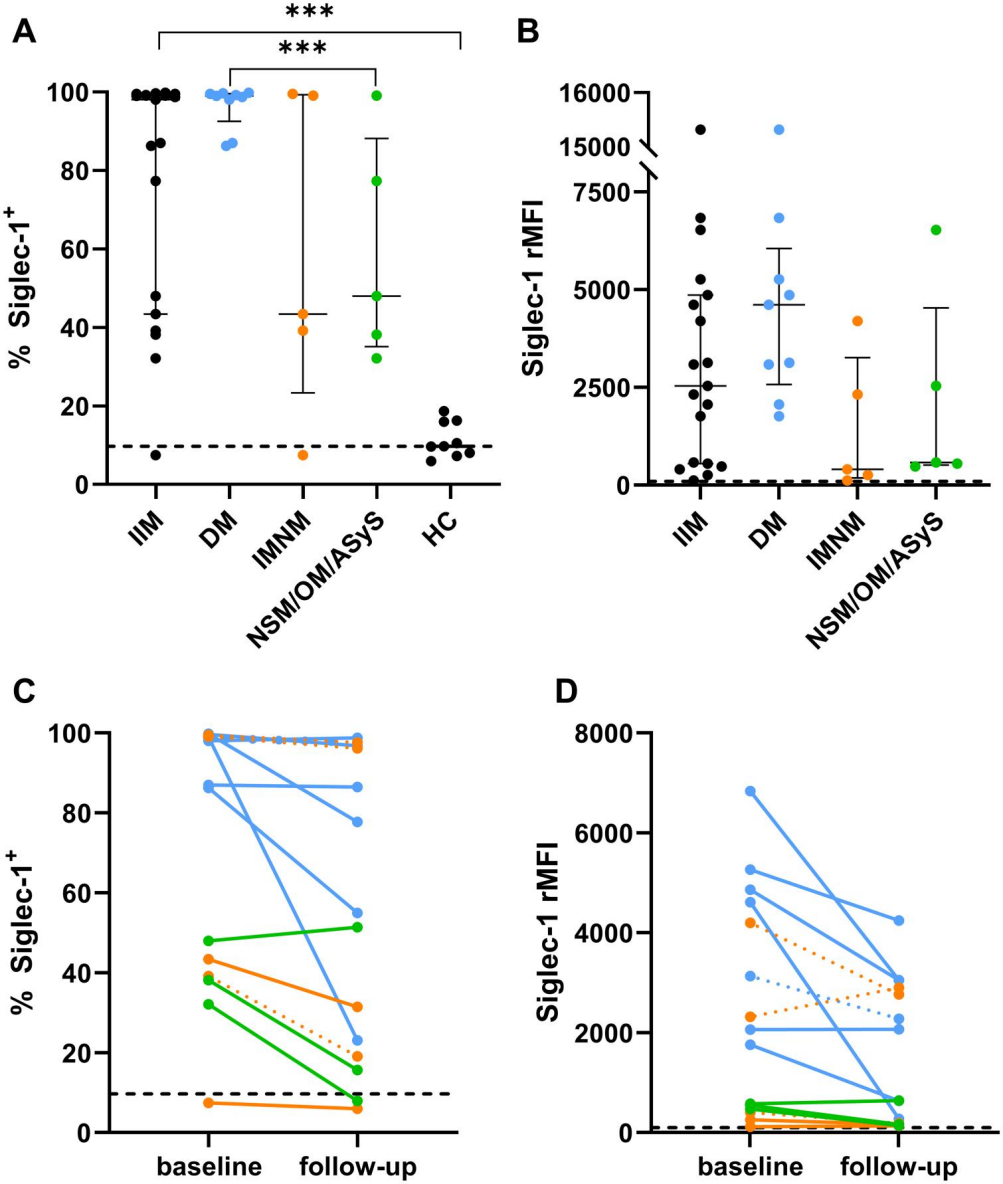
Siglec-1 expression in IIM patients and subgroups at baseline and follow-up. (**A**) Percentage of Siglec-1-positive CD14^+^ cells at baseline. (**B**) Siglec-1 rMFI at baseline. (**C**) Percentage of Siglec-1-positive CD14^+^ cells at baseline and follow-up. (**D**) Siglec-1 rMFI at baseline and follow-up. Panels (**C**) and (**D**) only show patients with complete baseline and follow-up data. Blue, DM; orange, IMNM; green, NSM/OM/ASyS. Dashed line in panel (**A**) represents median value of HC. Dashed line in panel (**B**) represents median MFI in HC, set at level 100. Connecting lines in panels (**C**) and (**D**) represent individual patients, of which the dotted lines represent patients with a shorter follow-up than 9 weeks (*n* = 4; sampling after 3 weeks in one DM and one IMNM patient; sampling after 6 weeks in two IMNM patients). Siglec-1, sialic acid-binding immunoglobulin-like-lectin-1; rMFI, relative median fluorescence intensity; IIM, idiopathic inflammatory myopathies; DM, dermatomyositis; IMNM, immune-mediated necrotizing myopathy; NSM/OM/ASyS, non-specific myositis/overlap myositis/antisynthetase syndrome; HC, healthy controls

At baseline, Siglec-1 expression was increased in IIM patients compared with HC, both in % of Siglec-1-positive CD14+ cells (median 98.1%, IQR 43.4–99.2 vs median 9.7%, IQR 7.6–16.2, *P* < 0.001; Fig. 2A) and rMFI (median 2541, IQR 549–4865, median in HC set at 100; Fig. 2B). Comparison between IIM subgroups showed a higher % of Siglec-1 positive CD14+ cells in DM vs NSM/OM/ASyS (*P* = 0.019) and higher Siglec-1 rMFI in DM vs IMNM (*P* = 0.029), and not between other subgroups (Fig. 2B).

Very high Siglec-1 expression (rMFI 15317) was observed in one DM patient with anti-MDA-5 autoantibodies, without ILD. Out of four patients with IMNM, clearly elevated Siglec-1 expression levels (rMFI) were seen in two HMGCR-positive patients (MxA in muscle biopsy negative in both patients). Two patients in the NSM/OM/ASyS group showed clearly elevated Siglec-1 expression (rMFI); both patients had OM, with newly diagnosed MCTD and Sjögren’s syndrome, respectively.

At follow-up, the median percentage of Siglec-1-positive CD14^+^ cells decreased over time in the IIM group, and in the subgroups DM and IMNM (Fig. 2C, *P* = 0.008, *P* = 0.043 and *P* = 0.043, respectively). The median rMFI decreased in the IIM group and in the subgroup DM (Fig. 2D, *P* = 0.013 and *P* = 0.028, respectively). The percentage of Siglec-1-positive CD14^+^ cells and rMFI remained relatively high at follow-up, particularly in DM patients (median of 86.5% in DM).

Out of the 15 patients with follow-up samples, four patients prematurely ended the study due to an insufficient clinical response to IVIG monotherapy (DM (*n* = 1), IMNM (*n* = 3)). Except for one IMNM patient, three of the patients had increased Siglec-1 expression at baseline (>rMFI of 2.000) which remained increased at follow-up (Fig. 2C and D).

### Associations between Siglec-1 expression and disease activity

**Figure 3.**
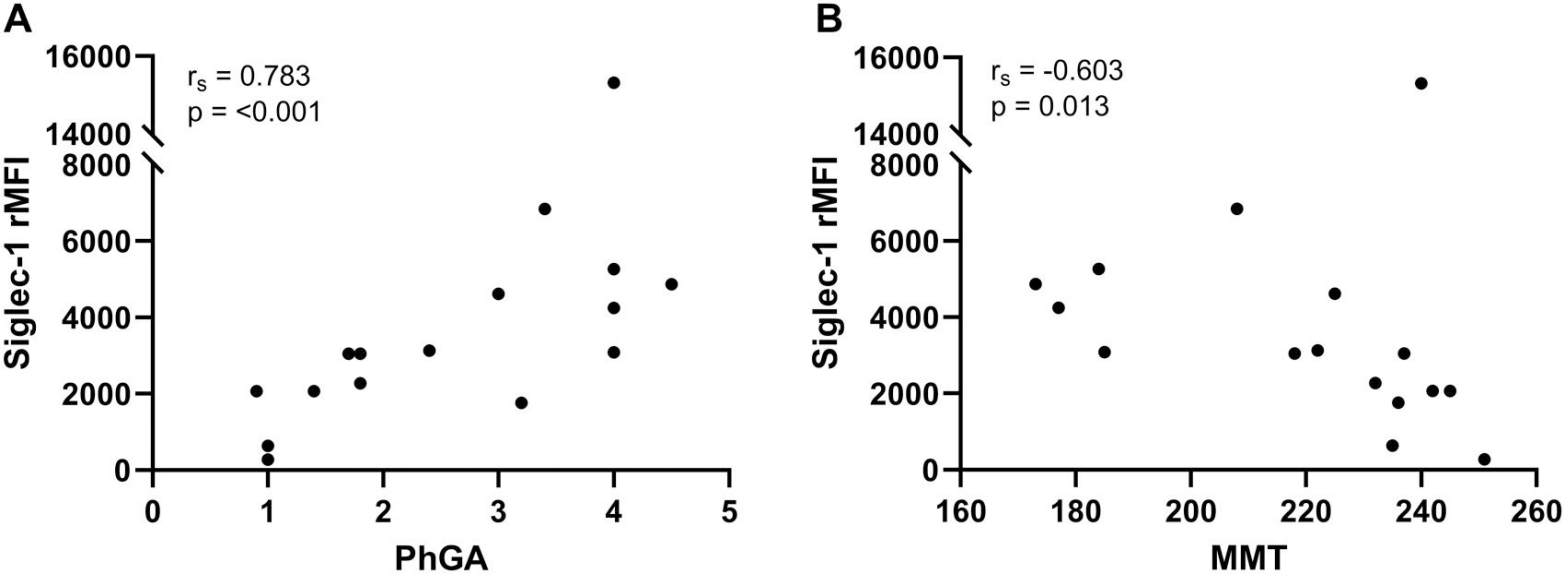
Correlation between Siglec-1 expression and PhGA and MMT in DM patients. (**A**) Spearman’s correlation between Siglec-1 rMFI and PhGA score. (**B**) Spearman’s correlation between Siglec-1 rMFI and MMT. Data include baseline and follow-up data. DM, dermatomyositis; Siglec-1, sialic acid-binding immunoglobulin-like-lectin-1; rMFI, relative median fluorescence intensity; PhGA, physician global assessment, score ranging from 0 (no disease activity) to 10 (severe disease activity); MMT, manual muscle testing, score ranging from 0 (no strength) to 260 (maximum strength)

Within the IIM group, Siglec-1 rMFI correlated with the PhGA score (*r*_s_ 0.488, *P* = 0.003) and manual muscle strength (MMT) score (*r*_s_ = −0.442, *P* = 0.011). Both correlations were driven by DM (PhGA, *r*_s_ = 0.783, *P* < 0.001, Fig. 3A; MMT, *r*_s_ = −0.603, *P* = 0.013, Fig. 3B) and IMNM patients (PhGA, *r*_s_ = 0.894, *P* < 0.001; MMT, *r*_s_ = −0.697, *P* = 0.025). In IIM patients, no correlations between percentage of Siglec-1-positive CD14^+^ cells and clinical parameters were observed. In the subgroup of DM patients, the percentage Siglec-1-positive CD14^+^ cells correlated with the PhGA score (*r*_s_ = 0.788, *P* < 0.001) and the MMT score (*r*_s_ = 0.656, *P* = 0.006). Siglec-1 did not correlate with sCK in IIM or subgroups.

### Associations between Siglec-1 expression and treatment response

**Figure 4.**
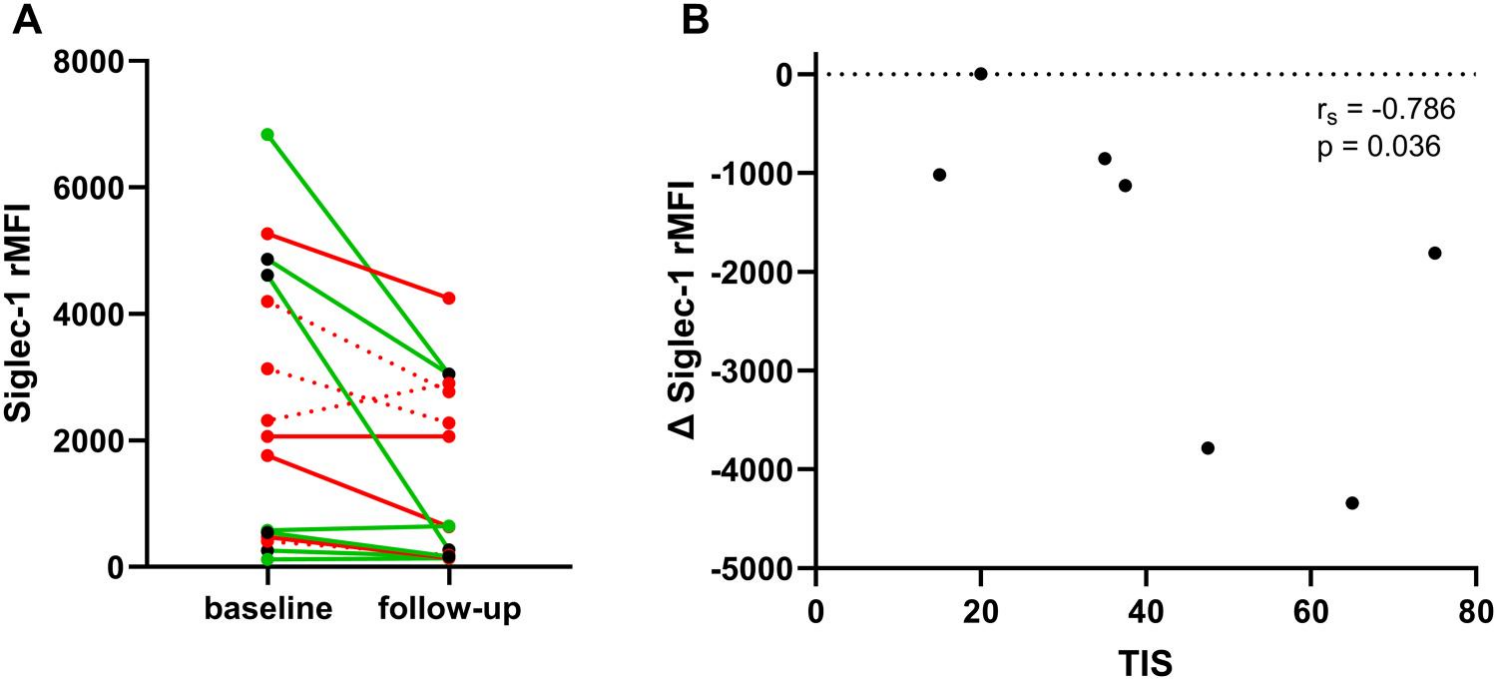
Siglec-1 and treatment response. (**A**) green: responders (TIS ≥ 40 points), red: non-responders (TIS < 40 points), the dotted lines represent patients with insufficient clinical response and a shorter follow-up than 9 weeks (*n* = 4; sampling after 3 weeks in one DM and one IMNM patient; sampling after 6 weeks in two IMNM patients). The black dots represent responders who met the criteria for major improvement (TIS ≥ 60). (**B**) Spearman’s correlation between change in Siglec-1 from baseline to follow-up and the TIS in DM patients. DM, dermatomyositis; IMNM, immune-mediated necrotizing myopathy; Siglec-1, sialic acid-binding immunoglobulin-like-lectin-1; rMFI, relative median fluorescence intensity; TIS, total improvement score

In the IIM group, change in Siglec-1 expression (delta rMFI) from baseline to follow-up did not correlate with the TIS (*r*_s_ = −0.242, *P* = 0.404), whereas in DM patients (*n* = 7), change in Siglec-1 did correlate with the TIS (*r*_s_ = −0.786, *P* = 0.036, Fig. 4A).

Change of Siglec-1 expression levels (rMFI) between baseline and follow-up appeared to be more pronounced in responders (TIS > 40; *n* = 7; green lines in Fig. 4B; 3/7 show a marked decline) as compared with non-responders (TIS < 40; *n* = 8; red lines in Fig. 4B), although this was not statistically significant (change scores: *P* = 0.662).

## Discussion

We found increased Siglec-1 expression in treatment-naive IIM patients, which declined in the majority of patients after nine weeks of treatment with IVIG monotherapy. Siglec-1 expression correlated with clinical disease activity in IIM (DM, and to a lesser extent in IMNM) and the change in Siglec-1 over time correlated with treatment response in DM. These clinical correlations with the IFN-I pathway emphasize its potential as a reliable biomarker of disease activity and treatment response, in particular in DM.

### Siglec-1 expression in DM

Our data extend findings by Graf *et al.* [17] who demonstrated increased Siglec-1 expression in newly diagnosed adult DM patients using a quantitative assay on whole blood samples. Notably, in their study two out of nine (22%) patients had normal Siglec-1 expression, which is in contrast with our cohort showing increased Siglec-1 levels in all DM patients, as measured on cryopreserved PBMCs. In the other study, information on treatment status at the time of sampling was not available. The difference in proportions of newly diagnosed adult DM patients with increased Siglec-1 expression may therefore be related to previous immunosuppressive treatment, or, alternatively, to differences in assays or differences in cut-offs based on healthy controls [17].

Our findings in adult DM align with studies in juvenile DM by others and our own group, showing increased Siglec-1 in treatment-naive newly diagnosed juvenile patients [16, 17]. Together, these data indicate that Siglec-1 is a sensitive marker of upregulation of the IFN-I pathway at diagnosis in (J)DM. In our cohort, no correlation was found between Siglec-1 and serum CK levels. Fifty-six percent of our DM patients had normal CK values at diagnosis, whereas Siglec-1 expression was upregulated in all DM patients. Given its low expression in healthy controls, our data suggest that Siglec-1 may have a value as a diagnostic marker, both confirming and ruling out the disease [25]. However, Siglec-1 may not differentiate between DM and other IFN-driven diseases or (viral) infections, and a threshold should be established for its clinical implementation.

One DM patient with anti-MDA-5 autoantibodies displayed remarkably high Siglec-1 level. The patient did not have ILD. Muscle biopsy was negative for MxA expression on immunohistochemical analysis. Graf *et al.* [17] also reported three DM patients (one of whom was anti-MDA-5^+^) with upregulated Siglec-1 levels in the blood but negative MxA expression on muscle biopsy. Presence of MxA in muscle has been observed by others in 71–77% of DM patients [4]. In particular, anti-MDA-5^+^ (J)DM patients have been reported to have no or weak MxA expression in muscle tissue, but relatively strong IFN-I signatures in blood and skin tissue [26–29]. This would be in line with the clinical phenotype of anti-MDA-5^+^ patients, who typically have limited muscle weakness with more severe extramuscular manifestations, such as interstitial lung disease and skin ulcerations [30].

An association between IFN-I gene expression and disease activity in DM patients has previously been established and could be corroborated using Siglec-1 in our study [7, 31]. Previous studies have shown correlations between Siglec-1 and the PhGA score in JDM and adult IIM [16, 17]. In the latter study, a correlation between Siglec-1 expression and PhGA was shown within a 3–12 month follow-up period, in a preselected group of patients. Our data confirm these findings, earlier in the disease in a more representative, albeit small group of patients. In combination with the results from Graf *et al.* [17] our findings underscore the clinical utility and feasibility of a blood cell-based biomarker for the IFN-I pathway in (J)DM. This approach is less time-consuming and/or methodologically challenging than analysis of a gene signature or the direct quantification of IFN-α or -β.

### Siglec-1 expression in IMNM

In IMNM, the involvement of the IFN-I pathway has, up to now, been thought to be limited. Marginal ISG expression was observed in muscle biopsies using RNA sequencing (e.g. 1.8 vs 101-fold increase of ISG15 in IMNM vs DM, respectively) and normal Siglec-1 expression was detected in whole blood samples of IMNM patients (*n* = 8, including juvenile patients and analyses of samples beyond 3 months since diagnosis) [6, 17]. Contrary to these findings, increased Siglec-1 expression (both in terms of % positive monocytes and rMFI) was observed in two out of five IMNM patients, who were newly diagnosed and treatment-naïve. Siglec-1 levels remained elevated at follow-up in two IMNM patients, which tended to correlate with limited response to therapy (early end-of-study due to insufficient response in both patients). It is notable that the increased Siglec-1 expression was not seen in the IMNM patient who had a concomitant connective tissue disease (CTD) but in two patients with autoantibodies against HMGCR and no signs of CTD. Larger cohort studies are needed to further examine to what extent, at diagnosis and during follow-up, the IFN-I pathway is upregulated in IMNM, and whether this reflects pathophysiology in muscle tissue, or an epiphenomenon. The latter seems less likely because Siglec-1 expression showed strong correlations with disease activity, including muscle strength, in our IMNM patients, similar to DM.

### Treatment response

In DM patients, Siglec-1 levels declined within a period of nine weeks of treatment with IVIG monotherapy. Change in Siglec-1 expression strongly correlated with the TIS, which is the most widely accepted composite outcome measure for treatment response in IIM [20]. This substantiates the clinical value of IFN-I pathway upregulation in the earliest phase of the disease and the value of Siglec-1 being able to detect these dynamic changes. We could not corroborate a relation between higher Siglec-1 expression levels at baseline and the need for treatment intensification within the first six months (data not shown), as we have shown previously in JDM (data not shown) [16].

### Clinical relevance

Because of the involvement of the IFN-I pathway in IIM, there has been growing interest in targeting this pathway therapeutically. The clinical efficacy and safety of inhibiting the IFN-I pathway in IIM by JAK-inhibitors is currently being investigated in randomized clinical trials [32]. Case series of patients with refractory DM treated with JAK-inhibitors, primarily tofacitinib, have reported response rates of 83–100%, with improvement of skin and muscle, as well as extramuscular disease (ILD) [9, 10, 33, 34]. Moreover, monoclonal antibodies targeting IFN-α, IFN-β and the IFN-I receptor are currently being investigated in phase-III trials in IIM [35, 36].

### Strengths and limitations

Strengths of our study include a well-characterized patient cohort, both in terms of diagnosis and outcome assessment. Furthermore, it is the first Siglec-1 assessment in a treatment-naive cohort with inclusion of different IIM subtypes, allowing for between-group comparisons. Study limitations include the small sample size and loss to follow-up due to low sample quality. Secondly, our treatment protocol (IVIG monotherapy) is not standard initial therapy for IIM [37, 38] which normally consists of high-dose steroids and steroid-sparing agents in most patients. Other studies have shown suppression of IFN-pathways after treatment with corticosteroids; implicating an effect of standard treatment on Siglec-1 expression during follow-up [39, 40]. Therefore, future research addressing the impact of corticosteroids is recommended. Considering the increased use of IVIG in IIM, particularly in refractory DM and early in the disease course of IMNM [20, 41–43], these data can still be valuable for understanding IFN-mediated disease mechanisms and their relation to clinical parameters. Furthermore, these insights may contribute to future patient stratification in the context of targeted treatment with JAK-inhibitors [37, 44].

In conclusion, this study provides new insights in the dynamics of Siglec-1 in adult IIM. Demonstrating responsiveness during the early stages of the disease, Siglec-1 also reflects disease activity and treatment response, particularly in DM. A joined international effort is necessary to collect a large number of samples to substantiate these findings, learn about long-term dynamics of Siglec-1, its role in non-DM subtypes and its ability to predict treatment response.

## Data Availability

The data underlying this article are available from the corresponding author.
